# Dexamethasone Protects Neonatal Hypoxic-Ischemic Brain Injury via L-PGDS-Dependent PGD_2_-DP_1_-pERK Signaling Pathway

**DOI:** 10.1371/journal.pone.0114470

**Published:** 2014-12-04

**Authors:** Pablo J. Gonzalez-Rodriguez, Yong Li, Fabian Martinez, Lubo Zhang

**Affiliations:** Center for Perinatal Biology, Division of Pharmacology, Department of Basic Sciences, Loma Linda University School of Medicine, Loma Linda, California, 92350, United States of America; Universidade de São Paulo, Brazil

## Abstract

**Background and Purpose:**

Glucocorticoids pretreatment confers protection against neonatal hypoxic-ischemic (HI) brain injury. However, the molecular mechanism remains poorly elucidated. We tested the hypothesis that glucocorticoids protect against HI brain injury in neonatal rat by stimulation of lipocalin-type prostaglandin D synthase (L-PGDS)-induced prostaglandin D_2_ (PGD_2_)-DP_1_-pERK mediated signaling pathway.

**Methods:**

Dexamethasone and inhibitors were administered via intracerebroventricular (i.c.v) injections into 10-day-old rat brains. Levels of L-PGD_2_, D prostanoid (DP_1_) receptor, pERK1/2 and PGD_2_ were determined by Western immunoblotting and ELISA, respectively. Brain injury was evaluated 48 hours after conduction of HI in 10-day-old rat pups.

**Results:**

Dexamethasone pretreatment significantly upregulated L-PGDS expression and the biosynthesis of PGD_2_. Dexamethasone also selectively increased isoform pERK-44 level in the neonatal rat brains. Inhibitors of L-PGDS (SeCl_4_), DP_1_ (MK-0524) and MAPK (PD98059) abrogated dexamethasone-induced increases in pERK-44 level, respectively. Of importance, these inhibitors also blocked dexamethasone-mediated neuroprotective effects against HI brain injury in neonatal rat brains.

**Conclusion:**

Interaction of glucocorticoids-GR signaling and L-PGDS-PGD_2_-DP_1_-pERK mediated pathway underlies the neuroprotective effects of dexamethasone pretreatment in neonatal HI brain injury.

## Introduction

Perinatal hypoxia-ischemia (HI) brain injury is a leading cause of acute mortality and chronic disability in newborns with an incidence of 1–8 cases per 1000 births, ultimately affecting 60% of preterm infants, which causes long-lasting morbidity, including cerebral palsy, seizure, and cognitive retardation in infants and children [1], [2]. Unfortunately, no definitive therapeutic interventions are available for most kinds of neonatal HI brain injury nowadays except that several studies indicated the potential benefits of hypothermia in some mild or moderate cases [3], [4], which may be, at least in part, due to the incomplete understanding of the basic pathogenesis in neonatal HI brain injury.

It is well recognized that glucocorticoids are critically implicated in various pathological processes as well as the physiological regulation of growth and development [5]. Recent emerging evidence implied central roles of glucocorticoids in programming the vulnerability of fetal and neonatal brain to hypoxia-ischemia challenge [5]. Our recent studies also revealed that dexamethasone pretreatment confers neuroprotective effects and reverses maternal hypoxia exposure induced enhanced susceptibility to neonatal HI brain injury [6]. However, the underlying molecular mechanism remains to be elucidated.

Lipocalin-type prostaglandin D synthase (L-PGDS) was originally identified as an enzyme in the brain responsible for catalyzing the isomerization of PGH_2_, synthesized by cyclo-oxygenase (COX)-2, to produce PGD_2_, as well as functions as an extracellular transporter for lipophilic ligands such as retinoids, tyroids, retinoic acid and amyloid peptides [7]–[11]. Prostaglandin D_2_ is the most abundant prostaglandin in the brain which affects sleep, temperature and nociception chiefly through two distinct G protein-coupled receptors, DP/DP_1_ (D prostanoid) receptor and DP_2_/CRTH_2_ (chemoattractant receptor homologous expressed on Th_2_ cells) [12]–[14]. Recent emerging evidence has revealed the positive effects of L-PGDS/PGD_2_ mediated pathway in various pathological processes [15]–[17]. Herein, we present the evidence of a novel finding that dexamethasone pretreatment protects against hypoxic-ischemic brain injury via activation of L-PGDS-dependent PGD_2_-DP_1_ signaling in the neonatal rat brain, of which pERK-44 acts as the major downstream kinase effector.

## Materials and Methods

### Experimental animals

Female Sprague Dawley rats with 8-day-old neonates (P8) were purchased from Charles River Laboratories (Portage, MI). Pups of mixed sex from different litters were randomly divided into the following groups: (1). Saline control group, n = 18; (2). Dexamethasone group, n = 18; (3). PD98059 group, n = 11; (4). SeCl_4_ group, n = 11; (5). MK-0524 group, n = 11; (6). Dexamethasone + PD98059 group, n = 11; (7). Dexamethasone + SeCl_4_ group, n = 11; (8). Dexamethasone + MK-0524 group, n = 11. All rats were kept in a room maintained at 24**°**C, a 12-h light/dark cycle, and provided *ad libitium* access to normal rat chow and filtered water. All procedures and protocols were approved by the Institutional Animal Care and Use Committee of Loma Linda University and followed the guidelines by the National Institutes of Health Guide for the Care and Use of Laboratory Animals.

### Brain Hypoxic-Ischemic (HI) treatment

Functional studies were performed by inducing brain HI injury in P10 rat pups, using a modified Rice-Vannucci model, described previously [6], [18], [19]. In brief, pups were anesthetized with 2% isoflurane, a small incision was made in the right side of the neck where the right common carotid artery was exposed and ligated with silk surgical suture. The incision was sutured. After recovery for 1 hour, pups were treated with 8% O_2_ for 2 hours. Following 3 hours of recovery on a warm pad, pups were returned to their moms.

### Reagents treatment

To explore the molecular mechanism of dexamethasone induced neuroprotection, selective inhibitors of L-PGDS (SeCl_4_, Sigma-Aldrich; 5 mg/kg), MEK1 (PD98059, Sigma-Aldrich; 30 ug/kg) and the antagonist of DP_1_ receptor (MK-0524, Santa Cruz; 40 ug/kg) were used in presence and absence of dexamethasone (Sigma-Aldrich; 2 ug/kg). These chemical reagents were injected into the right lateral ventricle prior to the HI treatment. Pups were anesthetized and fixed on a stereotaxic apparatus (Stoelting). An incision was made on the skull surface and bregma exposed. Each reagent or a cocktail was injected at a rate of 1 ul/minute with a 10 ul syringe (Stoelting) on the right hemisphere following the coordinates relative to bregma: 2 mm posterior, 1.5 mm lateral and 3.0 mm below the skull surface [6], [18], [19]. Saline was injected as the vehicle control. The injection lasted 2 minutes and the needle was kept for additional 5 minutes before its removal. The incision was sutured, and the animals were allowed to recover on a 37°C heated blanket. The animals were returned to their dams after recovering from anesthesia.

### Infarct size measurement

Pups were anesthetized and euthanized 48 hours after the HI treatment. Brain infarct size was determined as previous described [6], [18], [19]. Coronal slices of the brain (2 mm thick) were cut and immersed in a 2% solution of 2,3,5-triphenyltetrazolium chloride monohydrate (Sigma-Aldrich) for 5 minutes at 37°C and then fixed by 10% formaldehyde overnight. Each slice was weighed, photographed separately, and the percentage of infarction area for each slice was analyzed by Image J software (Version 1.40; National Institutes of Health, Bethesda, MD), corrected by slice weight, summed for each brain, and expressed as a percentage of whole brain weight.

### Western immunoblotting

Brains were homogenized on an ice-cold lysis buffer containing 20 mM HEPES, 10 mM KCl, 1.5 mM MgCl2, 50 mM Tris-HCl, 10 mM EDTA, 0.1% Tween-20, 0.1% b-mercaptoethanol, 0.1 mM phenylmethylsulfonyl fluoride, 5 ug/ml leuptin and 5 ug/ml aprotinin, pH 7.4. Homogenates were centrifuged at 4°C for 10 min at 14,000 g, and supernatants aliquots were collected and stored at −80°C. Protein concentrations were determined using a protein assay kit from Bio-Rad. Samples with equal protein (40 µg) were loaded on 10% sodium dodecyl sulfate polyacrylamide gel (SDS-PAGE) and preformed the electrophoresis analysis. After electrophoresis, proteins were transferred to nitrocellulose membranes. Nonspecific binding was blocked in TBST containing 5% dry milk for 60 min at room temperature. The membranes were incubated with rabbit L-PGDS, DP_1_ (1∶200; Santa Cruz Biotechnology) and rabbit pERK1/2 (1∶1000; Cell Signaling) polyclonal antibody overnight at 4°C. The membranes were then washed and incubated with secondary horseradish peroxidase-conjugated goat anti-rabbit β-actin antibody (1∶4000; Santa Cruz Biotechnology). Protein bands were visualized with enhanced chemiluminescence reagents, and the blots were exposed to Hyperfilm (GE Healthcare). Results were analyzed and quantified by the Kodak electrophoresis documentation and analysis system with Kodak ID image analysis software. For comparison of the levels of L-PGDS, DP_1_ and pERK1/2 protein relative density between the groups, samples were normalized firstly to β-actin values and then presented as fold values relative to sham-treated animals.

### ELISA (Prostaglandin Quantification)

Forty-eight hours after dexamethasone treatment in the P10 pups, dexamethasone and control group were decapitated, the amount of PGD_2_ in their fresh-frozen right hemisphere brain was determined by ELISA [20], following the manufacturer’s instruction with slightly modification. This kit is based on the conversion of PGD_2_ to a stable methoxime derivative by treatment with methoxamine hydrochloride. Briefly, the right hemispheres were homogenized with 50 mM KH_2_PO_4_, 5 mM EDTA, 43 mM acetylsalicylic acid and pH 7.4 buffer. Homogenates were centrifuged at 13,600 g for 5 min and supernatants stored at −80**°**C. Samples were diluted with cold acetone, incubated on ice for 5 min and centrifuged at 3,000 rpm for 10 min. Frozen sample were lyophilized. PGD_2_ concentration was measured by a PGD-Methoxime (MOX) EIA kit (Cayman Chemicals).

### Statistical analysis

Data are expressed as mean ± SEM. Experimental number (n) represents neonates from different dams. Statistical significance (*p<0.05*) was determined by Student *t*-test between saline control and dexamethasone treatment groups.

## Results

### Dexamethasone up-regulates L-PGDS and PGD_2_ in the neonatal rat brain

**Figure 1:**
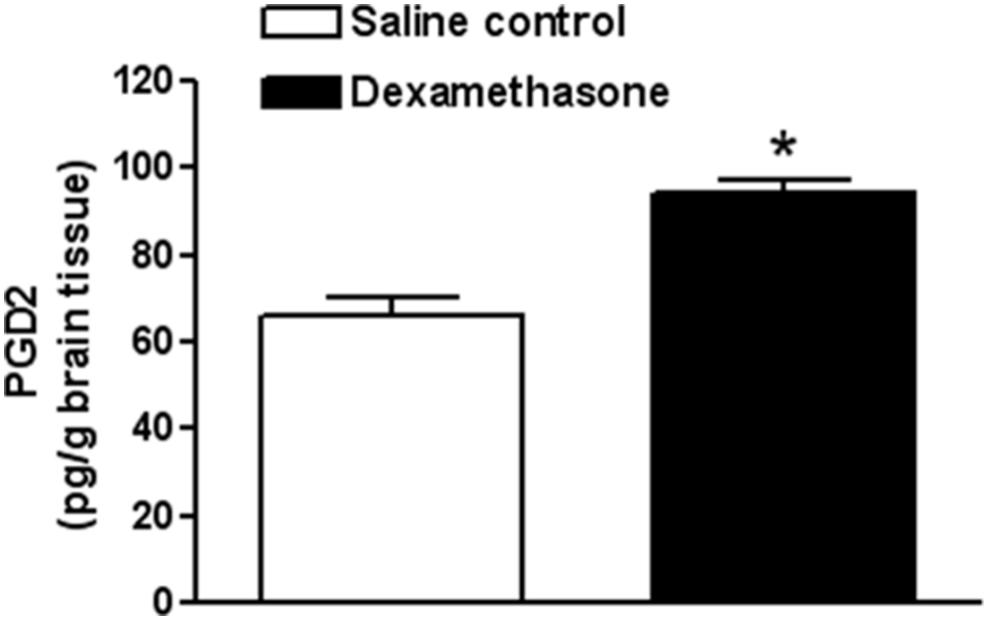
Effect of dexamethasone on expression level of PGD_2_ in neonatal rat brains. ELISA analysis demonstrated that dexamethasone pretreatment significantly up-regulated expression level of PGD_2_ in the developing rat brains. Data are mean ± SEM, n = 6. Statistical significance was determined by Student *t*-test between saline control and dexamethasone treatment groups. *p<0.05 dexamethasone versus saline control. PGD_2_ indicates prostaglandin D_2_.

**Figure 2:**
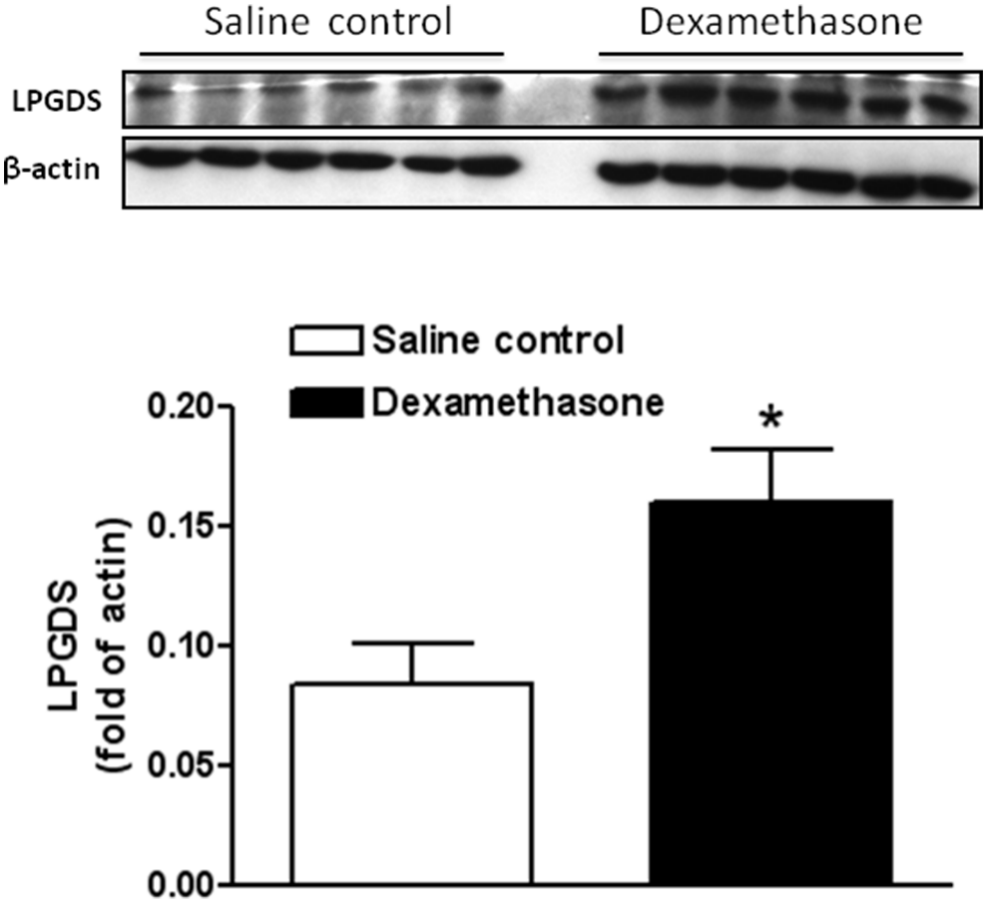
Effect of dexamethasone on protein abundance of L-PGDS in neonatal rat brains. Western blotting quantification showed that dexamethasone pretreatment significantly increased protein expression of L-PGDS in neonatal rat brains. Data are mean ± SEM, n = 6. Statistical significance was determined by Student *t*-test between saline control and dexamethasone treatment groups. *p<0.05 dexamethasone versus saline control. L-PGDS indicates lipocalin-type prostaglandin D synthase.

Firstly, we explored the potential effects of dexamethasone pretreatment on the expression profiles of the candidate proteins in the developing brain. We quantified expression levels of PGD_2_ in the neonatal rat brains 48 h after dexamethasone treatment by ELISA. The level of PGD_2_ was significantly up-regulated (*p<0.05 dexamethasone versus saline control; Figure 1) in the presence of the glucocorticoids treatment. The level of L-PGDS was also determined in the presence of dexamethasone via Western Immunoblotting. As shown in Figure 2, the protein expression level of L-PGDS was also significantly increased (*p<0.05 dexamethasone versus saline control) 48 h after dexamethasone treatment, which indicated that glucocorticoids can concomitantly stimulate production of L-PGDS and PGD*_2_* in the developing rat brain.

### Dexamethasone exerts no effect on DP_1_ receptor expression

**Figure 3:**
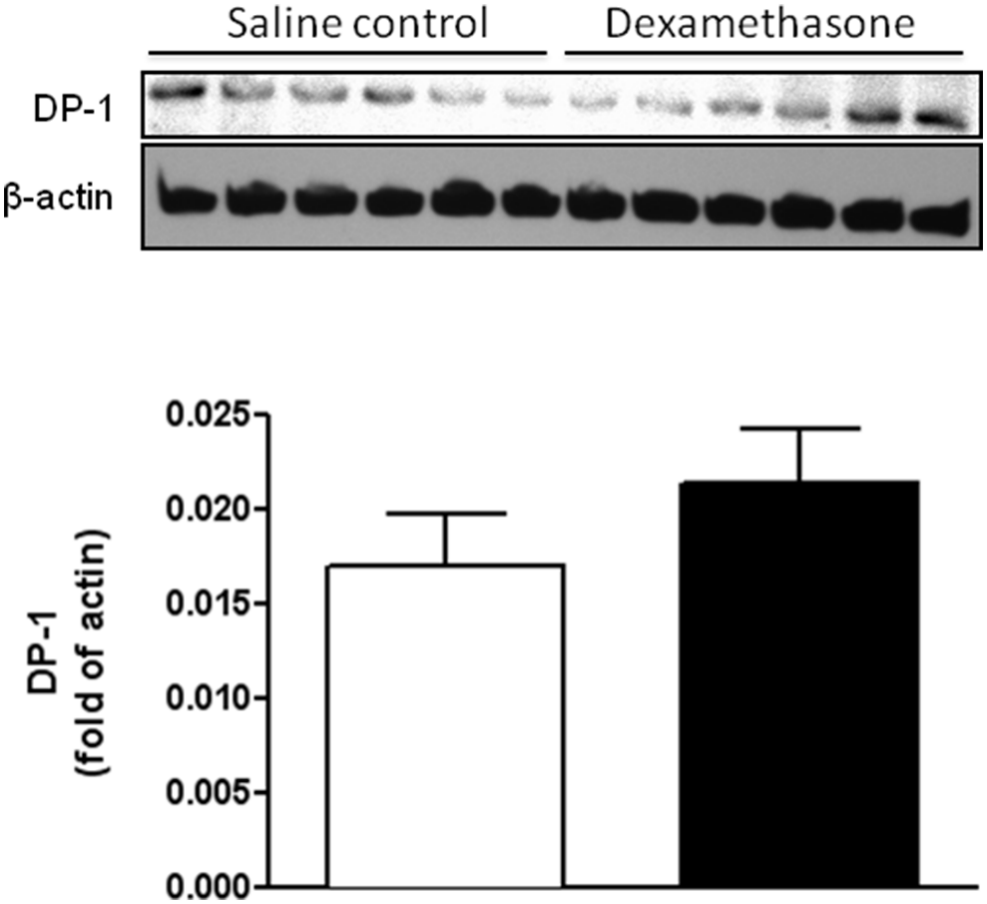
Effect of dexamethasone on protein abundance of DP_1_ receptor in neonatal rat brains. There was no significant difference of DP1 receptor expression observed in neonatal rat brains between control and dexamethasone treatment group. Data are mean ± SEM, n = 6. Statistical significance was determined by Student *t*-test between saline control and dexamethasone treatment groups. *p>0.05* dexamethasone versus saline control. DP_1_ indicates D prostanoid.

Recent evidence indicates that PGD_2_ functions as a protective mediator chiefly via activation of DP/DP_1_ (D prostanoid) receptor [15]–[17]. Thus, we determined whether dexamethasone pretreatment also affects expression levels of DP_1_ receptor in the developing brain. As shown in Figure 3 by western blotting analysis, no significant difference was observed between control and dexamethasone treatment group (*p>0.05* dexamethasone versus saline control).

### Dexamethasone increases pERK-44 in L-PGDS-PGD_2_-DP_1_ dependent manner

**Figure 4:**
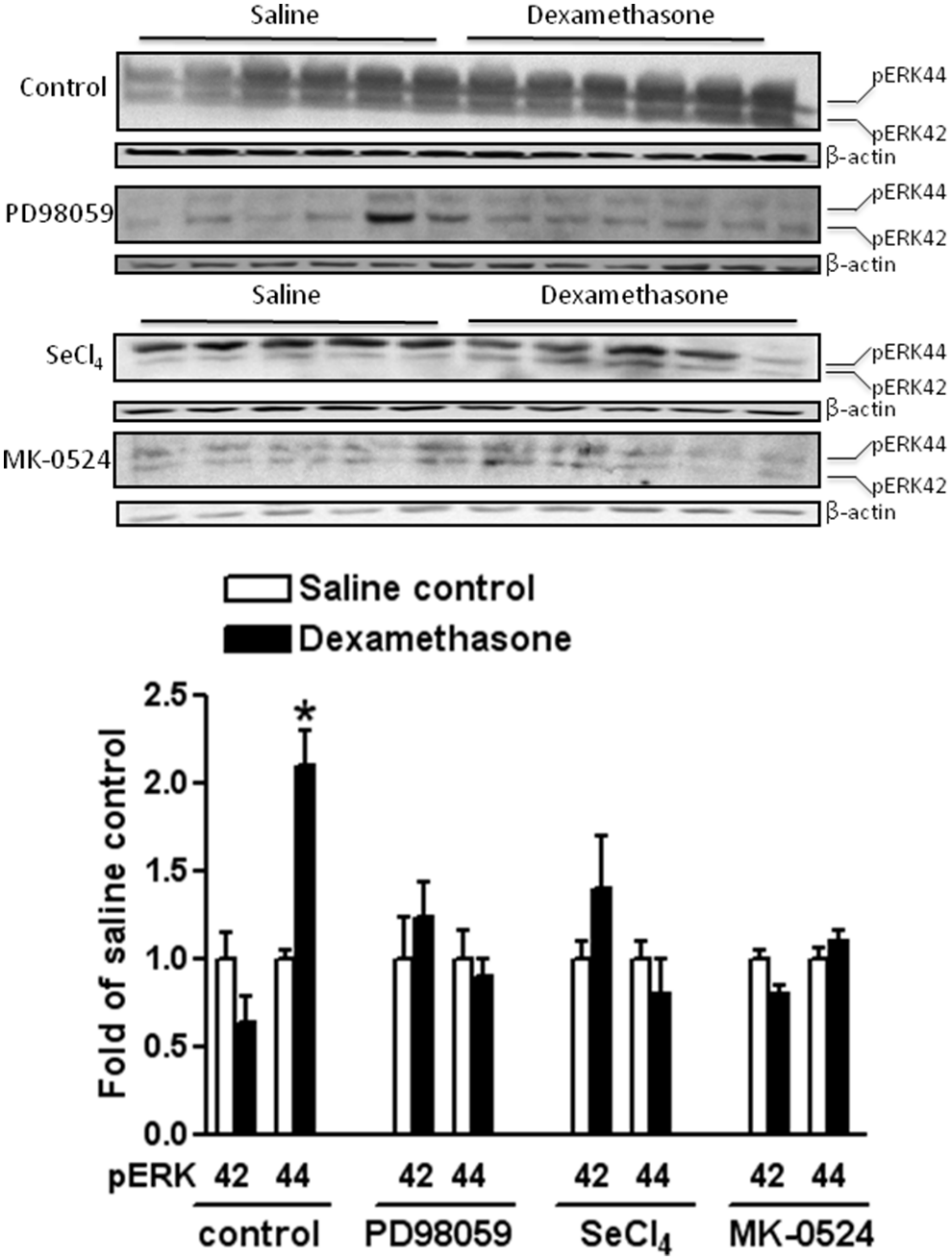
Inhibitory effects of PD98059, SeCl_4_ and MK-0524 on dexamethasone-induced expression of pERK-42/44 in neonatal rat brains. Western blotting quantification indicated that dexamethasone pretreatment selectively enhanced expression level of pERK-44 in neonatal rat brains. However, such effects were inhibited by PD98059, SeCl_4_ and MK-0524, respectively. Up-regulation of pERK-44 by dexamethasone pretreatment chiefly relied on activation of L-PGDS-PGD_2_-DP_1_ signaling pathway in the developing rat brains. Data are mean ± SEM, n = 5 each group. Statistical significance was determined by Student *t*-test between saline control and dexamethasone treatment groups. *p<0.05 dexamethasone versus saline control. SeCl_4_ indicates selenium chloride, a selective inhibitor of lipocalin-type prostaglandin D synthase (L-PGDS); PD98059, a selective inhibitor of mitogen-activated kinase/ERK kinase 1(MEK1); MK-0524, a selective antagonist for prostaglandin D_2_ receptor (DP_1_); pERK-42/44 indicates phosphorylated extracellular signal regulated kinase 42/44.

It has been well documented that ERK-42/44 confers protective property in various physiological and pathological processes in the brain, which might function as a common major downstream effector in signaling transductions [21], [22]. In the present study, we further evaluated the expression levels of pERK-42/44 in the presence of dexamethasone. Interestingly, dexamethasone pretreatment selectively increased the expression of pERK-44 (*p<0.05 dexamethasone versus saline control; Figure 4) in the neonatal rat brains, while there was no effect of the dexamethasone treatment on the expression of isoform pERK-42 (*p>0.05* dexamethasone versus saline control; Figure 4). Importantly, the enhanced expression levels of pERK-44 were abrogated by co-treatment with inhibitors of L-PGDS (SeCl_4_), MEK1 (PD98059) and the antagonist of DP_1_ receptor (MK-0524), respectively (Figure 4), which suggested that up-regulation of pERK-44 by dexamethasone pretreatment chiefly relied on activation of L-PGDS-PGD_2_-DP_1_ signaling pathway in the developing rat brain.

### Dexamethasone attenuates neonatal HI brain injury via activation of L-PGDS-PGD_2_-DP_1_-pERK-44 mediated pathway

**Figure 5:**
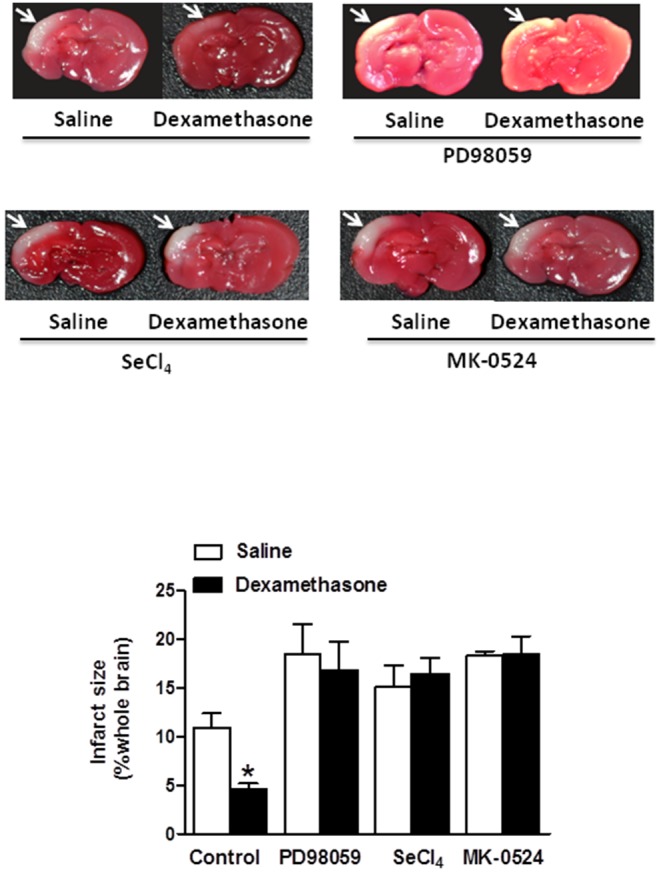
Blocking effects of PD98059, SeCl_4_ and MK-0524 on dexamethasone pretreatment-induced neuroprotection against hypoxic-ischemic (HI) brain injury in P10 rat pups. Dexamethasone was injected into the right lateral ventricle in day 10 (P10) pups before HI-induced brain injury in the absence or presence of the MEK1 inhibitor (PD98059), L-PGDS inhibitor (SeCl_4_) or DP_1_ antagonist (MK-0524). Infarct size at the ipsilateral brain hemisphere was measured *via* TTC staining (shown by arrows), and reported as percent of the whole brain. Data are mean ± SEM, n = 6 each group. Statistical significance was determined by Student *t*-test between saline control and dexamethasone treatment groups. *p<0.05 dexamethasone versus saline control.

**Figure 6:**
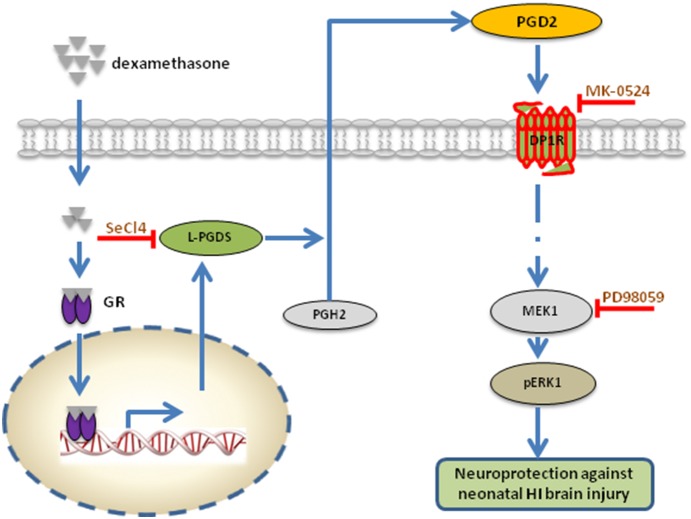
Proposed signaling pathway in dexamethasone pretreatment-induced neuroprotection against neonatal hypoxic-ischemic (HI) brain injury. Dexamethasone interacts with the glucocorticoids receptor (GR) in the cytoplasm, promotes the translocation of GR into the nucleus, and increases L-PGDS expression, which stimulates conversion of PGH_2_ to PGD_2_. PGD_2_ then binds to DP_1_ receptor (DP_1_R) and initiates a series of intracellular signaling pathways, of which MEK1/pERK1 function as the major downstream effectors. Upregulation of pERK1 confers dexamethasone pretreatment-induced neuroprotective effects in neonatal HI brain insult. Inhibition of L-PGDS, PGD_2_ or MEK1 *via* correspondent inhibitors (SeCl_4,_ MK-0524 or PD98059) thus inhibits dexamethasone-induced neuroprotection, suggesting the interaction of glucocorticoids/GR and L-PGDS-PGD_2_-DP_1_-pERK signaling pathway underpinning the neuroprotective effect of dexamethasone on neonatal HI brain injury. GR: glucocorticoid receptor; L-PGDS: lipocalin-type prostaglandin D synthase; PGH_2_: prostaglandin H_2_; PGD_2_: prostaglandin D_2_; DP_1_: D prostanoid; MEK1: mitogen-activated kinase/ERK kinase 1; pERK-42/44: phosphorylated extracellular signal regulated kinase 42/44; SeCl_4_: selenium chloride, a selective inhibitor of lipocalin-type prostaglandin D synthase (L-PGDS); PD98059: a selective inhibitor of MEK1; MK-0524: a selective antagonist for prostaglandin D_2_ receptor (DP_1_).

We further determined the functional consequences of dexamethasone pretreatment in the neonatal HI brain injury in the absence or presence of the MEK1 inhibitor (PD98059), L-PGDS inhibitor (SeCl_4_) or DP_1_ antagonist (MK-0524). HI-induced infarct size in the neonatal rat brain was analyzed *via* TTC staining. As shown in Figure 5, the dexamethasone pretreatment demonstrated a neuroprotective effect and decreased HI insult-induced infarct size of neonatal rat brains from 10.95±1.46% to 4.56±0.65% (*p<0.05). This neuroprotective effect was blocked in the presence of PD98059 (16.77±3.00% versus 18.47±3.03%, *p>0.05*), SeCl_4_ (16.38±1.70% versus 15.10±2.25%, *p>0.05*), or MK-0524 (18.36±1.34% versus 18.32±0.42%, *p>0.05*) (Figure 5). Taken together, these functional studies suggested a novel molecular mechanism that interaction of glucocorticoids-GR and L-PGDS-PGD_2_-DP_1_-pERK signaling pathway underpins the neuroprotective effects of dexamethasone pretreatment on neonatal HI brain injury (Figure 6).

## Discussion

Our present study presents several novel findings: 1. Dexamethasone pretreatment significantly stimulated L-PGDS-dependent dominant PGD_2_ biosynthesis in the neonatal rat brain; 2. PGD_2_ up-regulation accounted for the dexamethasone pretreatment induced neuroprotective effects against neonatal HI brain injury via activation of DP_1_ receptor; 3. pERK-44 acted as the major downstream effector in PGD_2_-DP_1_ receptor mediated signaling pathway; 4. at the molecular level, interaction of glucocorticoids-GR signaling and L-PGDS-PGD_2_-DP_1_-pERK pathway afforded the protection of dexamethasone pretreatment against neonatal HI brain injury.

As described previously [5], brain is one of major targets of glucocorticoids, which induce various physiological and pathological effects chiefly via activation of glucocorticoid receptor (GR). Of interest, glucocorticoids demonstrate both neurodegenerative and neuroprotective effects in variant brain pathologies [23]. A large amount of evidence also indicated the bidirectional effects of glucocorticoids in the context of various similar brain insults including neonatal HI brain injury, which relied on experimental protocol, dosage, time, animal age, strains and species [5], [24]–[29]. It may be partly due to the incomplete understanding of fundamental mechanisms of glucocorticoids action and inappropriate designs of administration. Our recent study revealed that maternal hypoxia exposure represses GR expression in the developing brain, resulting in heightened susceptibility to neonatal HI brain injury, while pretreatment with dexamethasone, a selective GR agonist, confers neuroprotective effects and reverses maternal hypoxia induced pathological process, which confirmed the pivotal role of GR actions in the brain development and pathology [6]. Consistently, our present study reproduced those positive effects of dexamethasone pretreatment in neonatal HI brain injury, further revealing the pivotal roles of glucocorticoids-GR signaling in the pathogenesis of developing brain and enforcing the justification to clarify its underlying molecular mechanisms.

In the present study, dexamethasone treatment increased expression levels of both L-PGDS and PGD_2_, which suggested a novel interaction between glucocorticoids-GR signaling and the arachidonic acid cascade. L-PGDS functions as an isomerase in the brain to catalyze isomerization of PGH_2_ and preferentially promote biosynthesis of PGD_2_. It had been reported recently that dexamethasone pretreatment could stimulate the common pathway for dominant prostaglandin biosynthesis by up-regulating of cPLA_2_-COX_2_ expression as well as concomitant production of L-PGDS through genomic effects of GR activation in cardiomyocytes [17]. How the GR activation also co-induces L-PGDS and PGD_2_ biosynthesis in the developing brain, which warrants further exploration. Another problem is that two types of PGDS have been purified and characterized: lipocalin-type PGDS (L-PGDS) and the hematopoietic PGDS (H-PGDS), both mediate the last regulatory steps in the biosynthetic pathway of PGD_2_ production [30], [31]. Whether dexamethasone pretreatment induces H-PGDS expression in the developing brain and contributes to PGD_2_ production, also deserving future investigation. Emerging evidence has indicated the protective effects of L-PGDS in neuropathology. One study reported that L-PGDS is the most abundant cerebrospinal fluid protein produced in the human brain and might function as a major endogenous chaperone to prevent the aggregation of Abeta [32]. L-PGDS-deficient mice exhibited an exacerbated phenotype following transient or permanent ischemic brain injury, indicating a critical role of L-PGDS in protection against cerebral ischemia [33]. Recently, it has been documented that L-PGDS protected against neuronal cell death due to oxidative stress and might function as an early stress protein to protect against HI brain injury in a mouse model [34], [35]. In addition, dexamethasone treatment via GR activation can induce L-PGDS mRNA and protein expression in mouse neuronal cells [36]. In the present study, we observed that inhibition of L-PGDS by SeCl_4_ abrogated dexamethasone pretreatment induced neuroprotective effects in neonatal rat brains with HI insult, which not only confirmed its protective role in various brain injuries but also indicated its key role in glucocorticoids-GR signaling mediated neuroprotection.

Both *in-vitro* and *in-vivo* studies have demonstrated a neuroprotective role for PGD_2_ [15], [16]. In our present study, dexamethasone pretreatment induced protective effects in HI brain injury was blocked by pharmacologic antagonizing DP_1_ receptor with MK-0524. It suggested that PGD_2_ mediated dexamethasone-induced protective effects against HI brain injury via mainly stimulation of DP_1_ receptor. Two PGD_2_ receptors had been identified: the DP_1_ and the DP_2_ receptor, which stimulates adenylyl cyclase through G_αs_ or inhibits adenylyl cyclase through G_αi_ and increases intracellular Ca^2+^, respectively [37]–[39]. Our study implied that dexamethasone induced L-PGDS expression, preferentially promoted PGD_2_ biosynthesis in the developing brain, which acted as a local mediator in an autocrine and/or paracirne manner to confer protective effects via chiefly interaction with DP_1_ receptor (Figure 6). However, we cannot totally exclude the effects of DP_2_ receptor activation based on the data available in present study. In addition, PGD_2_ is relatively unstable and readily metabolized to the more stable product, such as J series of prostanoids, which confer anti-inflammatory and antioxidant effects via activation of PPARγ [40]. A recent study had already revealed that prostaglandin D_2_ and its metabolites protect the heart against ischemia-reperfusion injury by activating Nrf2 via FP receptor, proposed another novel mechanism by which glucocorticoids to afford cardioprotection [41], which may be also applicable in the present study.

Another finding of the present study is that pERK-44 acted as the major downstream effector in dexamethasone pretreatment mediated neuroprotection in neonatal HI brain injury. pERK-42/44 can function as the major downstream kinase accounting for dexamethasone-induced cardioprotection in PGD_2_-DP_1_ signaling pathway in cardiomyocytes [17]. Our study replicated such effects but further in the setting of the developing brain with HI insult *in vivo*. Interestingly, we observed that dexamethasone selectively increased expression of isoform pERK-44 but no effect on pERK-42. In vitro studies, where morphine selectively down-regulated pERK-42 levels with no effect on pERK-44, have also demonstrate a selective regulation on pERK1/2 [42]. One in vitro study reported that pERK induces phosphorylation of Thr125 in caspase-9 via a targeted conserved MAPK consensus site to block the caspase-9 processing and subsequent caspase-3 activation, conferring anti-apoptotic effects [43]. pERK mediated protective effects had been well identified in various physiological and pathological conditions, including the neonatal HI brain injury [21], [22]. Several literatures also documented that pERK can be induced by glucocorticoids-GR signaling in certain context. One recent research reported that GR activation by stress induced glucocorticoids enhances contextual fear memory via tPA-BDNF-TrkB-Erk1/2 signaling pathway [44], which is not contradictory to our findings in the present study, of which dexamethasone pretreatment increases pERK in an L-PGDS-PGD_2_-DP_1_ dependent manner, but further confirmed the diverse effects of glucocorticoids-GR signaling changing with various settings.

Our present study reveals a novel molecular mechanism underlying the glucocorticoids mediated protective effects in the setting of neonatal HI brain injury, the interaction between glucocorticoids-GR signaling and L-PGDS dependent PGD_2_-DP_1_-pERK pathway, which would significantly contribute to both the basic and clinic study. Perinatal HI brain injury is still a major cause of death and disability worldwide. Although there are multiple advances in research of cellular processes and molecular mechanism, hypothermia is the only effective treatment in certain neonatal HI cases at present. To combination of hypothermia with neuroprotective interventions seems to be a promising way to improve the neurological outcome of HI. Glucocorticoids have been widely administered in various clinic settings. However, its effectiveness remains to be controversial, especially in the context of certain brain diseases including neonatal HI injury, which may be ascribed to the inappropriate administration designs but also due to incomplete appreciation of its fundamental mechanisms. Our study reveals another novel but clinical relevant mechanism of glucocorticoids action in HI brain injury at the molecular level which would not only shed new insights on the pathogenesis of HI brain injury but also offer novel promising interventional molecular targets and contribute to the well-being of HIE in newborns.
